# Preparation of Lignin Nanoparticles from Wood Waste for Wood Surface Treatment

**DOI:** 10.3390/nano9020281

**Published:** 2019-02-17

**Authors:** Florian Zikeli, Vittorio Vinciguerra, Alessandro D’Annibale, Donatella Capitani, Manuela Romagnoli, Giuseppe Scarascia Mugnozza

**Affiliations:** 1 Department for Innovation in Biological, Agro-Food and Forest Systems (DIBAF), University of Tuscia, Via S. Camillo Lellis, snc, 01100 Viterbo, Italy; zikeli@unitus.it (F.Z.); vincigue@unitus.it (V.V.); dannib@unitus.it (A.D.); mroma@unitus.it (M.R.); 2 Magnetic Resonance Laboratory “Annalaura Segre” Istituto di Metodologie Chimiche, Consiglio Nazionale delle Ricerche (IMC-CNR), Area della Ricerca Roma 1, Via Salaria km 29.300, 00015 Monterotondo, Italy; donatella.capitani@cnr.it

**Keywords:** lignin, nanoparticles, NMR spectroscopy, Iroko, coatings

## Abstract

Lignin was isolated from wood wastes comprising Iroko sawdust (IR) and mixed sawdust from Iroko and Norway spruce (IRNS), furnished by a local wood houses producer. The respective acidolysis lignin fractions were structurally characterized using pyrolysis (Py)-GCMS, two-dimensional heteronuclear single quantum correlation nuclear magnetic resonance (2D HSQC NMR), Fourier-transform infrared FTIR and ultraviolet-visible (UV-VIS) spectroscopies, size exclusion chromatography, and standard wet-chemistry methods for Klason lignin and polysaccharides determination. The isolated lignin fractions were subsequently used for the preparation of lignin nanoparticles (LNPs) using a non-solvent method. LNPs were then used for wood surface treatment using a dip-coating technique. The coated wood samples were analyzed by colorimetry and scanning electron microscopy (SEM) before and after artificial weathering experiments in a UV chamber to investigate the UV protection potential of the LNPs coatings. Wood samples dip-coated with LNPs showed promising surface modifications resembling a sort of film of fused LNPs. Coatings made from IR-LNPs and IRNS-LNPs performed significantly better in artificial weathering experiments than uncoated reference samples.

## 1. Introduction

In addition to playing a structural role in higher plants, lignin provides a protective shield against weathering effects, via making cell walls hydrophobic and thus preventing their swelling in wet conditions, and against the attack of pathogens by creating a compact lignified wood tissue non-penetrable by microorganisms [1,2]. That concept might serve as inspiration to develop new bio-based formulas for the protection of wood and other materials. Formulations for the protection and preservation of wood surfaces often contain toxic substances and their long-term impact on human health has to be considered when destined to interiors furniture [3,4,5]. Furthermore, their industrial production itself might have negative effects on our environment as well [6,7]. The design of new protection strategies relying on lignin, especially when isolated from wood wastes, could contribute strongly to the realization of an ideally circular economy. Since lignin is the most abundant renewable feedstock for aromatic compounds with UV-absorbing properties [8], it deserves special attention when thinking of wood-protection agents against UV radiation. From this point of view, lignin was successfully incorporated into cellulose films resulting in a 100% and 90% protection from UV-B (280–320 nm) and UV-A (32–400 nm) radiation, respectively [9]. Furthermore, the supplementation of either lignin or lignin nanoparticles (LNPs) to commercial sunscreen formulations led to a significant increase in their sun protection factors [10,11]. However, as lignin is also considered as the main player of photodegradation of wood [12,13], efficacy and durability of a wood-protection layer based on pure lignin need to be thoroughly investigated. Nevertheless, in the opinion of the authors a wood-protection formulation based on lignin could provide a strong but probably temporal shield against weathering. Consumption of the protective shield over time could however be encountered with regeneration of the coatings by repeated applications of the product based on widely available lignin.

Wood wastes such as sawdust are generated as a side-stream in saw mills at amounts of around 50% of processed timber and are currently used mostly for energy generation with revenues as low as 26 € per ton of wood biomass [14]. This high material loss needs to be addressed even more when working with certain high-value woods used for their outstanding properties but being imported over long distances. In these cases, the development of new wood waste-based products, instead of energy generation, should be considered as a major priority in wood industries in order to lower transport costs as well as decreasing the environmental impact of their overall production process through the implementation of new value-chains [15]. The present study focused on two wood wastes, namely the sawdust from Iroko (*Milicia excelsa* (Welw.) C.C. Berg) and mixed sawdust from Iroko and Norway spruce (*Picea abies* (L.) H. Karst.). These byproducts represent two parallel waste streams in the respective production processes of an Italian wood houses producer (Pagano Costruzioni in Legno, Rome, Italy) where laminated timber from Norway spruce is superficially finished with Iroko sawn timber to improve its durability, shorten maintenance periods as well as for static and esthetic reasons. Iroko wood, as it was found also for other tropical wood species [16], has been long known for its outstanding resistance to decay, which is reportedly due to its extractives content, chlorophorin in particular [17,18].

In this study, acidolysis lignin (AL) fractions, isolated from sawdust of Iroko as well as from mixed sawdust of Iroko and Norway spruce, were structurally characterized prior to their use for the preparation of LNPs by a non-solvent technique [19]. Then, aqueous dispersions of LNPs were used for a surface treatment of beech wood via dip-coating and the stability to artificial weathering was tested. To this aim, the coated samples as well as homologous non-coated samples underwent an artificial weathering treatment in a UV chamber which is the most common test of resistance to weathering degradation. LNPs or colloidal lignin particles have been already used to tune commercial wood stains [20]. However, and to the best of our knowledge, this is the first study reporting on the use of pure LNPs dispersions as a single agent for wood-protection applications against weathering.

## 2. Materials and Methods 

Iroko (*Milicia excelsa* (Welw.) C.C. Berg) sawdust (IR) and mixed sawdust from Iroko and Norway spruce (*Picea abies* (L.) H. Karst.) (IRNS) were collected during wood processing at the facility of a wood houses producer (Pagano Costruzioni in Legno, Rome, Italy). The sawdust was air-dried and then milled to 35 mesh in an IKA MF 10.1 cutting mill (IKA®-Werke GmbH & Co. KG, Staufen, Germany). The milled wood (15 g) was subjected to Soxhlet-extraction using acetone (400 ml) for 15 h followed by drying at room temperature. The removal of aromatic extractives compounds is necessary for a correct consecutive lignin quantitative analysis and structural characterization.1,4-Dioxane (Alfa Aesar, ACS-grade 99+%) was purchased from Thermo Fisher (Kandel) GmbH, Karlsruhe, Germany. ACS-grade sodium hydroxide and dimethyl sulfoxide (DMSO) (>99.9%,) were purchased from Carlo Erba reagents (Milan, Italy) and Sigma-Aldrich (Milan, Italy), respectively.

### 2.1. Lignin Extraction and Preparation of Lignin Nanoparticles (LNPs)

Acidolysis lignin fractions (AL-IR and AL-IRNS) were isolated from the acetone-extracted sawdust samples of Iroko and Iroko–Norway spruce using a modified protocol of Gellerstedt et al. [21], as described in our recent publication [19].

LNPs were prepared following a modified protocol of Lievonen et al. [22], treating initial lignin-solutions in DMSO with water as anti-solvent using dialysis tubes made of regenerated cellulose, as described in Zikeli et al. [19]. The resulting LNP dispersions (LNPs-IR, LNPs-IRNS) were recovered and kept in the fridge until use. Additionally, for the consecutive dip-coating LNPs fractions from beech and chestnut AL, prepared as described in Zikeli et al. [19], were used. 

### 2.2. LNPs-Coating of Beech Wood Samples

Beech wood samples of tangential cuts with a size of 20 × 20 mm and a thickness of 3–4 mm were sanded with abrasive paper (grain number 220) and processed by a dip-coating protocol adapted from Herrera et al. [23]. First, the samples were immersed for 5 min into aqueous dispersions of LNPs (concentration: 3.5 mgmL^−1^) during that the dispersion was soaked up by the wood samples. Then, three cycles (1 min) of full immersion in distilled water were applied to wash away non-soaked-up excessive LNPs dispersion. Afterwards the samples were left in a 60 °C drying oven for complete evaporation of the water and complete deposition of the LNPs on and inside the wood cell wall elements. This procedure represented one layer of LNPs-coating and was repeated four times to create a total of 5 layers of LNPs-coating. For all four LNPs-samples (IR, IRNS, B, Ch) four specimens were prepared for the accelerated weathering tests in the UV chamber.

### 2.3. Accelerated Weathering Testing

The LNPs-coated wood samples were subjected to artificial weathering in a Q-LAB accelerated weathering tester (model: QUV/se, Q-LAB Corporation, Westlake, OH, USA) using UVA-340 lamps with a spectrum simulating “outdoor” conditions continuously irradiating at 1 Wm^−2^nm^−1^ at 340 nm and 50 °C and a relative humidity of 20% at 50 °C. Coated and control samples, four repetitions each, were left in the UV chamber for 1, 3, 5 and 7 days and their CIELAB color space was analyzed throughout the weathering treatment time using a Konica Minolta CM-700d Spectrophotometer colorimeter (Konica Minolta Sensing Europe B.V., Nieuwegein, Netherlands). The overall color difference was calculated according to

$$\Delta E=\sqrt{\Delta L^{2}+\Delta a^{2}+\Delta b^{2}}$$

where Δ*E* is the color difference, *L* is the lightness, *a* is the red-green coordinate and *b* is the yellow-blue coordinate.

### 2.4. Analytical Methods

Extractives contents of the different wood species were determined gravimetrically after acetone extraction for 15 h using a Soxhlet apparatus. Klason lignin (KL), cellulose and hemicellulose contents of wood as well as KL contents of AL samples were determined gravimetrically according to TAPPI T 222 om-02 [24] and the methods of van Soest [25] and van Soest and Wine [26], respectively.

### 2.5. FTIR and UV Spectroscopy

FTIR spectra of the isolated AL fractions and LNPs (after drying of the dispersions) were recorded on a FTIR-4100 Fourier Transform Infrared spectrometer (Jasco Corporation, MD, USA). After very fine grinding of the samples in an agate mortar, potassium bromide (KBr) discs were prepared with a lignin concentration of 5% (wt.) using a Specac Mini-Pellets-Press (Specac Inc., Fort Washington, MD, USA). The spectra were acquired in the absorbance mode in the range of 4000–400 cm^−1^ with a resolution of 4 cm^−1^ against a background of pure KBr. 

UV-VIS spectra of the AL and LNP fractions in Milli-Q water, which was used as blank, were recorded on a V-630 spectrophotometer in the range of 200–400 nm (Jasco Corporation, MD, USA).

### 2.6. Analytical Pyrolysis

Pellets of fine-powdered wood, lignin and acidolysis residues (1.5–2.0 mg) were pressed in a special syringe and eventually directly pyrolyzed at 450 °C in a Pyrojector II (SGE) microfurnace pyrolysis chamber. Pyrolysis products were separated in a HP 5890 Series II Plus gas chromatograph endowed with a Restek Rtx®-1701 (30 m × 0.25 mm i.d., 0.25 µm film thickness) capillary column. Helium was the carrier gas at a pressure of 100 kPa in the pyrolyzer and 70 kPa in the GC injector (280 °C, 1:20 split ratio). The temperature in the oven was held initially at 45 °C for 4 min, then increased to 240 °C with a heating rate of 4 °C min^−1^ and finally until 280 °C at a rate of 39°Cmin^−1^. A HP 5971A-MSD mass spectrometer was used in EI mode at 70 eV and scans from *m*/*z* 35 to *m*/*z* 500 were run in 0.7 s cycles. Pyrolysis products were identified by mass spectra interpretation and by comparison with NIST and Wiley computer libraries and reference literature [27,28,29,30,31]. For each pyrogram, the relative area of 31 principal phenolic pyrolysis products was calculated, compared to the most intense peak, and grouped within those derived from either G-type or S-type lignin to determine the S/G ratio.

### 2.7. Two-Dimensional NMR Spectroscopy

Two-dimensional heteronuclear single quantum coherence (2D HSQC) NMR spectra were recorded on a Bruker AVANCE III 600 MHz spectrometer with a z-gradient probe using 55 mg of the lignin samples in 700 µL DMSO-d_6_. The HSQC experiments were acquired using a time domain of 1024 data points in the F2 dimension (^1^H) and 512 data points in the F1 dimension (^13^C). A *^1^J_C-H_* coupling constant of 150 Hz and spectral widths of 11.5 ppm and 200 ppm in the ^1^H- and ^13^C-dimensions, respectively, were used applying 128 scans with a recycle delay of 3 s. For a semi-quantitative analysis, the integrals of the HSQC cross-peaks were determined and compared using MestReNova software (v6.0.2, Mestrelab Research S.L., Santiago di Compostela, Spain). Signal assignment was done according to literature [17,32,33,34,35,36,37,38,39]. The monomeric ratio of the AL fractions was estimated from the C_2_-H_2_ correlations from S, G, and H units in the aromatic region of the HSQC spectra. The C*_α_*-H*_α_* correlations were used for the relative abundances of the different lignin inter-unit linkages as well as the ratio of cinnamyl aldehyde end groups.

### 2.8. Molar Mass Distribution

Molar mass distributions of the lignin samples were investigated by alkaline high performance-size exclusion chromatography (HP-SEC) using a PSS MCX column with 5 µm particle size and 1000 Å pore size (PSS Polymer Standard Services, Mainz, Germany) equilibrated with 10 mM NaOH with 20 mM sodium nitrate (NaNO_3_) at a flow rate of 0.6 mLmin^−1^ on a Varian high-performance liquid chromatograph (HPLC) system with UV detection at 280 nm. The concentration of the lignin samples and the injection volume were 3 mgmL^−1^ and 10 µL, respectively. Calibration for molar mass determination was done with sodium polystyrene sulfonate reference standards (PSS Polymer Standard Services, Mainz, Germany) with the following molar masses at peak maximum (M_p_): 65400 Da, 33500 Da, 15800 Da, 6430 Da, 1670 Da, 891 Da, and 208 Da.

### 2.9. SEM of LNPs and LNPs-Coated Beech Wood

For scanning electron microscopy (SEM), the LNPs-coated samples were attached to aluminum stubs using carbon tape and sputter-coated with gold in a Balzers MED 010 unit. SEM analysis was conducted with a JSM 6010LA electron microscope (JEOL Limited, Tokyo, Japan). Size measurements of LNPs deposited on the wood surfaces were conducted on the SEM micrographs using the Adobe Photoshop CS4 Extended software package (Adobe Systems, San Jose, CA, USA). SEM experiments were carried out on samples before and after artificial weathering, according to the different steps.

## 3. Results and Discussion

### 3.1. Acidolysis Lignins—Isolation and Structural Characteristics

**Figure 1:**
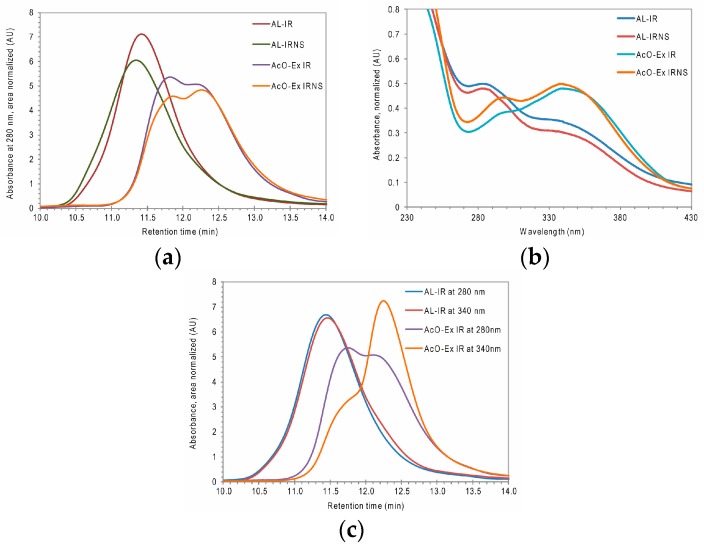
(**a**) HP-SEC elution profiles at 280 nm of the acidolysis fractions from Iroko and mixed sawdust of Iroko and Norway spruce (AL-IR and AL-IRNS) as well as their respective acetone-extractives fractions (AcO-Ex IR and AcO-EX IRNS); (**b**) UV-VIS spectra (230–430 nm range) of AL and AcO-Ex fractions from IR and IRNS; (**c**) HP-SEC elution profiles at 280 nm vs. 340 nm of AL-IR and AcO-EX IR.

IR and IRNS sawdust showed very low hemicelluloses contents and lignin contents around 30% KL. Acidolysis reduced the hemicelluloses content almost to zero and delivered AL fractions with a purity of nearly 90% KL (Table 1). Isolation yields were lower than those previously observed for beech wood waste or chestnut sawdust [19], albeit they can still be considered as representative. Both AL-IR and AL-IRNS showed similar, rather high molar mass averages, AL-IRNS with slightly higher values for M_w_ and M_n_, and uniform molar mass distributions (Table 1 and Figure 1a).

Considering the aromatic nature of some compounds suspected in the acetone-extractives fraction, the respective fractions from IR and IRNS were also analyzed by HP-SEC (AcO-Ex IR and AcO-EX IRNS in Figure 1a). The elution curves of both acetone-extractives fractions showed the presence of two close maxima where the first one strongly overlapped with the respective AL elution curves while the second one was eluted at a molar mass range of a few hundred Dalton. The comparison of the respective UV spectra of the two fractions shows a maximum at 340 nm for the acetone extractives and a corresponding shoulder peak in the UV spectrum of the ALs (Figure 1b). Overlapping of the HP-SEC elution curves at a wavelength of 280 nm delivered a first hint that certain lignin fractions supposedly were isolated together with the acetone extractives. This was further supported by comparing the elution curves at 280 nm with the respective curves at 340 nm of the acetone-extractives fractions (Figure 1c). There the peak eluting first has a stronger absorbance at 280 nm while the second eluting peak shows much stronger absorbance at 340 nm. Similarly, the HP-SEC curve of AL-IR shows a peak tailing which is stronger absorbing at 340 nm than at 280 nm wavelength.

**Figure 2:**
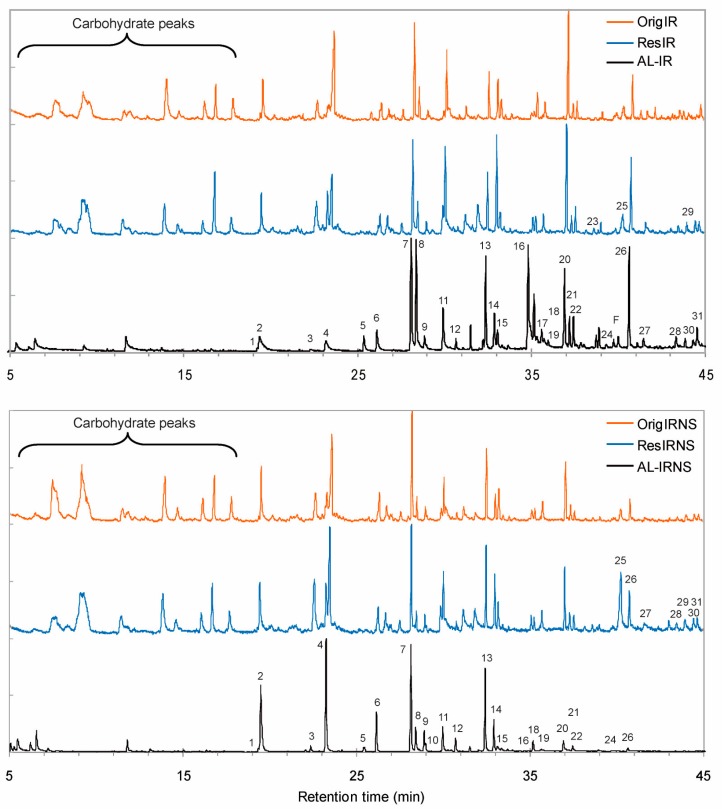
Pyrograms of Acidolysis lignin from Iroko and Iroko-Norway spruce sawdust (AL-IR, AL-IRNS), the respective initial sawdust samples (OrigIR, OrigIRNS) and the respective acidolysis residues (ResIR, ResIRNS).

The peaks in the pyrograms in Figure 2 with retention times lower than 19 min as well as peak 25 (levoglucosane) represent polysaccharides degradation products which are strongly abundant in the respective pyrograms of the original sawdust samples and the acidolysis residues (OrigIR, OrigIRNS, ResIR, ResIRNS). Polysaccharides pyrolysis products were described in detail for Corsican pine elsewhere [40]. In the pyrograms of both lignin samples (AL-IR, AL-IRNS), instead, those peaks are present in very low amounts confirming the purity of the AL samples suggested by their respective KL contents (Table 1). The pyrolysis products of AL-IR with the highest peak integrals were 4-vinylguaiacol, 4-propenylsyringol, resorcinol and 4-vinylphenol (peak numbers 7, 24 + 26, 16 and 18 in Figure 2 and Table 2) where resorcinol is supposed to derive from chlorophorin units. 4-vinylphenol is expected to derive, beside from regular lignin H units, also from *p*-coumaric acid units which were found in significant amounts based on the respective 2D NMR cross-peaks (Table 4). Moreover, 4-vinylphenol could derive from a dehydroxylated derivate of chlorophorin which was reported by Christensen et al. [17]. The most prominent peaks in the pyrogram of AL-IRNS were 4-methylguaiacol, 4-vinylguaiacol, guaiacol and isoeugenol (peak numbers 4, 7, 2 and 12 + 13 in Figure 2 and Table 2). In comparison to AL-IR, resorcinol and 4-vinylphenol appeared only in low amounts and the calculated S/G ratio was considerably lower (i.e., 0.18). That result can be attributed to the second component in this sawdust feedstock, namely to Norway spruce, which is a conifer with almost pure G-type lignin and low extractives content. Considering this, a future lignin isolation process with targeted lignin composition could be designed simply by blending different sawdust feedstock lines deriving from different wood species.

Compared to the original wood, the S/G ratios calculated for the isolated AL fractions were lower than those of the original wood samples (OrigIR and OrigIRNS) while the S/G ratios for the acidolysis residues (ResIR and ResIRNS) were higher for both feedstocks (Table 2). Similar findings were reported in our recent work [19] and they were explained by the fact that S-type lignin is generally deposited in deeper layers of the cell wall while G-units are rather found in middle lamella and primary cell wall regions [41]. Hence, the AL fractions showed a lower S/G ratio than the original wood because the part of S-type lignin in deeper cell wall regions was not accessible by the isolation method used here.

**Figure 3:**
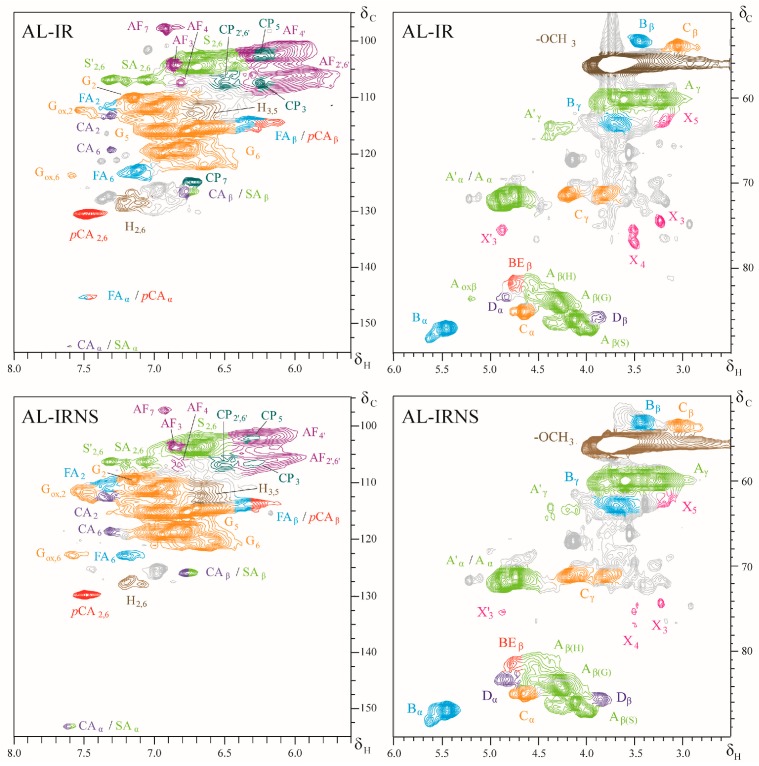
2D HSQC NMR spectra, aromatic (left) and side-chain regions (right) of acidolysis lignin from Iroko sawdust (AL-IR) and mixed sawdust from Iroko and Norway spruce (AL-IRNS).

**Figure 4:**
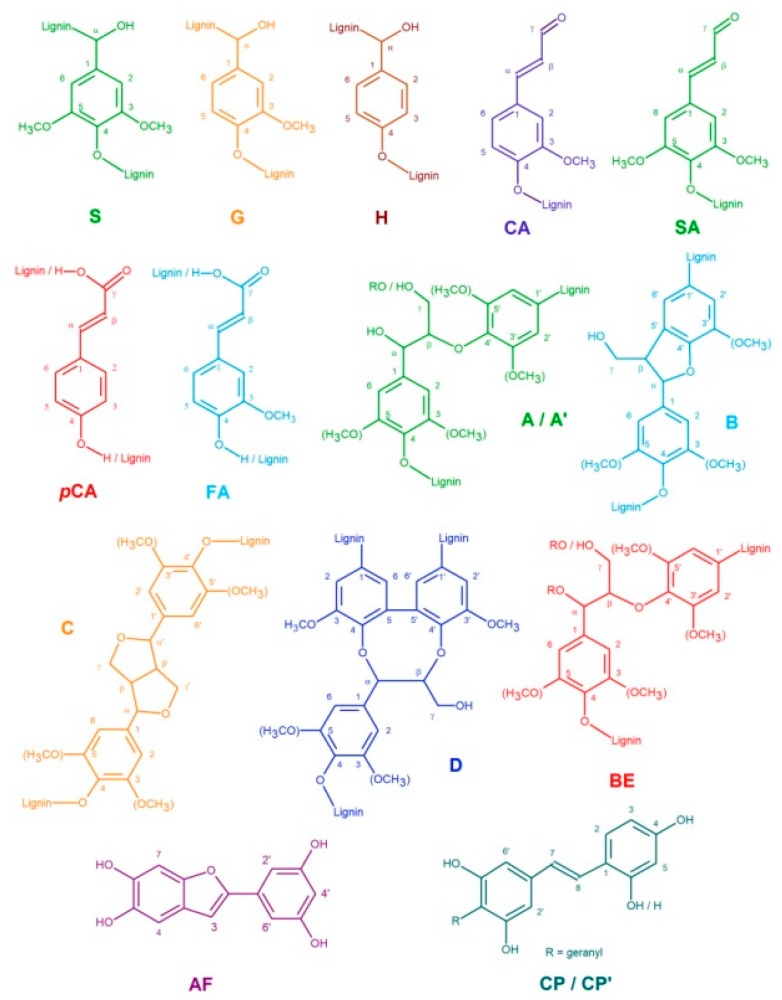
Lignin structural elements assigned to the respective cross-peaks in the 2D NMR spectra: syringyl (S), guaiacyl (G), *p*-hydroxyphenyl (H), coniferyl aldehyde (CA), sinapyl aldehyde (SA), *p*-coumaric acid (*p*CA), ferulic acid (FA), alfafuran (AF) and chlorophorin (CP, CP′) units; *β*-*O*-4′ (A) and *γ*-acylated *β*-*O*-4′ (A′) substructures, *β*-5′ phenylcoumaran (B), *β*-*β′* resinol (C), dibenzodioxocin (D) and benzyl ether (BE) substructures.

For more detailed structural characterization of the isolated AL fractions, two-dimensional HSQC NMR experiments were conducted. In Figure 3 the expanded aromatic and side-chain regions of the recorded 2D NMR spectra of AL-IR and AL-IRNS, respectively, are illustrated. Table 3 reports the chemical shifts of the cross-peaks that are assigned to their respective lignin structural elements, shown in Figure 4. For both samples AL-IR and AL-IRNS cross-peaks representing S and G-units are dominating the aromatic regions of the 2D NMR spectra. However, also cross-peaks for *p*-hydroxycinnamic acids, as *p*-coumaric (*p*CA) and ferulic acid (FA), were identified, as well as for cinnamyl aldehyde end groups (CA, SA). In the region of 100 ppm < δ_C_ < 110 ppm and 5.8 < δ_H_ < 6.4 ppm cross-peaks were registered that could not be assigned to regular lignin S-type structures which are typically found in those regions. Iroko wood is known to contain the stilbenoid chlorophorin and based on the reported spectral data, the cross-peaks for the aromatic carbon atoms C_3_ (δ_C_/δ_H_ = 107.5/6.46 ppm) and C_5_ (δ_C_/δ_H_ = 102.8/6.32 ppm) as well as C_2′_ and C_6′_ (δ_C_/δ_H_ = 106.9/6.16), involved in the stilbenoid structure, could be identified in the recorded 2D NMR spectra of AL-IR and AL-IRNS [17,42]. Although chlorophorin is expected to be removed during the initial extensive acetone extraction of the sawdust feedstocks, both 2D NMR and Py-GCMS (see above) data suggest the residual presence of chlorophorin in the isolated lignin fractions. Considering the presence of multiple phenolic hydroxyl groups and double bonds in chlorophorin structure, its incorporation into the lignin macromolecule via radical coupling might not be ruled out. Ouete et al. [43] isolated from Iroko leaves beside others a compound with phenylcoumaran structure, known also as alfafuran. Based on the reported ^1^H NMR data from Noguchi et al. [36] as well as comparison with spectral data for similar structures recently reported to be incorporated in lignin macromolecules [33], alfafuran was suspected as a component in the lignin fractions AL-IR and AL-IRNS. Furthermore, in the side-chain regions of the AL NMR spectra xylan cross-peaks were identified. 

Table 4 lists the estimated monomeric compositions of the isolated AL fractions as well as the calculated S/G ratios and relative abundances of different lignin inter-unit linkages. S and G-units are present almost equally in AL-IR (S/G ratio: 0.86) while H units are found only in low amounts of 5%. AL-IRNS instead consists to almost ¾ of G-units and around ¼ of S units resulting in an S/G ratio of 0.30, considerably lower than in pure Iroko caused by significant contribution of the softwood Norway spruce. The relative abundance of H units in AL-IRNS was with 3% even lower than for AL-IR. Comparing the results based on Py-GCMS and on 2D NMR spectroscopy, the two methods showed high accuracy for AL-IR, while for AL-IRNS the deviation between the two methods was slightly higher, but still representing the strong difference between the two lignins’ compositions.

Both AL fractions showed high ratios of *β*-*O*-4′-linkages indicating strong structural preservation of the native lignin using mild acidolysis for lignin isolation. Beside the main lignin inter-unit linkages, namely *β*-*O*-4′-, *β*-5′- and *β*-*β*′ structures, also signals for dibenzodioxocin (DBDO) and benzyl ether substructures were identified with low presence of 2–4% (Table 4). AL-IRNS showed a higher ratio of *β*-5′ substructures which related to the higher content of G-units arising from the softwood Norway spruce, where the C_5_ in the aromatic ring is free for inter-unit linkages.

**Figure 5:**
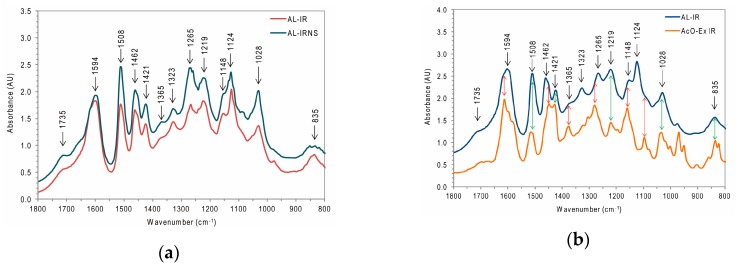
(**a**) FTIR spectra of the isolated acidolysis lignin fractions from Iroko and Iroko–Norway spruce sawdust (AL-IR, AL-IRNS); (**b**) FTIR spectrum of AL-IR compared to the spectrum of the respective acetone-extractives fraction from Iroko sawdust (AcO-Ex IR).

Regarding their S/G ratios, the difference between AL-IR and AL-IRNS was also visible based on the IR bands’ intensities in their respective FTIR spectra (Figure 5a) where for AL-IRNS the gap between I_1462_ (absorbance intensity at 1462 cm^−1^) and I_1508_ was much bigger than for AL-IR and the band at 1270 cm^−1^ (considered as a typical G lignin band) was much more prominent in the spectrum of AL-IRNS [21]. Moreover, when comparing the FTIR spectra of AL-IR and the respective acetone extractives (AcO-Ex IR in Figure 5b), several overlapping bands were identified (green double-arrows in Figure 5b at 1508 cm^−1^, 1421 cm^−1^, 1219 cm^−1^ and 1028 cm^−1^), indicating the presence of above mentioned aromatic compounds such as chlorophorin or alfafuran in the acetone-extractives fraction. Strong IR bands in the spectrum of AcO-Ex IR with slightly shifted maxima compared to the respective IR bands in the spectra of the isolated AL fractions and thus resulting in shoulder peaks there (red arrows in Figure 5b) deliver further indications for the co-presence of these aromatic acetone extractives in the AL fractions. Detailed FTIR band assignments can be found in the literature [13,44,45].

### 3.2. LNPs from Iroko and Iroko–Norway Spruce Sawdust

**Figure 6:**
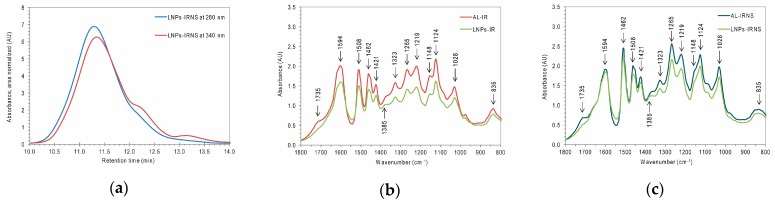
(**a**) HP-SEC elution curves of the dried lignin nanoparticles fractions from mixed sawdust re-dissolved in 10 mM NaOH (LNPs-IRNS) at 280 and 340 nm; (**b**,**c**) FTIR spectra of the Acidolysis lignin fraction from Iroko and Iroko–Norway spruce sawdust (AL-IR, AL-IRNS) and of their respective freeze-dried lignin nanoparticles (LNPs-IR, LNPs-IRNS).

Another piece of evidence for this conclusion can be found comparing the HP-SEC curves of the freeze-dried LNPs from IR and IRNS (Figure 6a) at different wavelengths (280 nm vs. 340 nm). The elution profile of LNPs from IRNS is characterized by higher absorbance values at 280 than at 340 nm up to a certain retention time value (around 12 min), starting from which the elution of low molecular weight fractions begins (Figure 6a). From that retention time onwards, the UV absorbance gets higher at 340 nm wavelength reflecting the stronger presence of extractives compounds in lignin macromolecules of the corresponding molar mass. Considering that the preparation of LNPs involved the use of a dialysis membrane with a molecular weight cut-off of 6–8 kDa, low molar mass compounds were expected to be removed. Consequently, a covalent bond between extractives compounds, as chlorophorin and alfafuran, and the lignin macromolecule might be suggested. 

The freeze-dried LNPs fractions were also analyzed by FTIR spectroscopy and their spectra strongly overlapped with the respective FTIR spectra of the pristine AL fractions, delivering evidence for the high structural preservation of the prepared lignin nanoparticles (Figure 6b,c). The only significant change in the spectra was a new band in the LNPs spectra at 1385 cm^−1^ that was already earlier observed for B-LNPs and Ch-LNPs in our recently published work [10] and attributed to a change in the chemical environment of cellulose residues on the lignins as reported by Colom et al. [13].

### 3.3. LNPs-Coated Wood—General Morphological Characteristics

**Figure 7:**
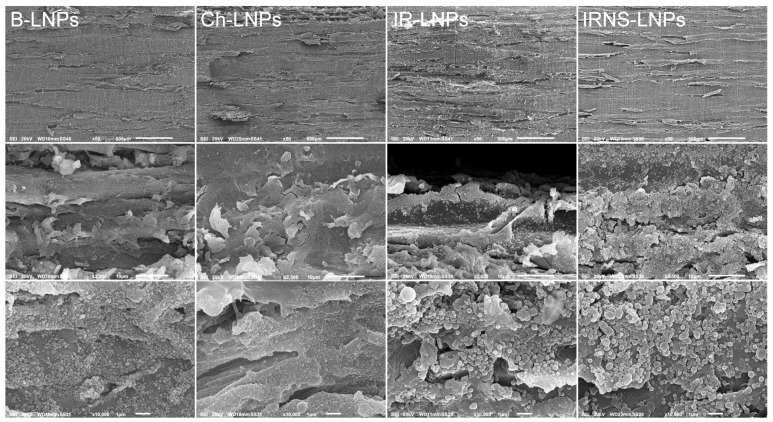
SEM micrographs at different magnifications (150×, 2200–2500×, 10000×) of the beech wood surfaces coated with LNPs from beech, chestnut, Iroko, and mixed Iroko–Norway spruce (B-LNPs, Ch-LNPs, IR-LNPs, IRNS-LNPs).

The micrographs obtained by SEM in Figure 7 show the beech wood surfaces dip-coated by LNPs from beech (B), chestnut (Ch), IR, and IRNS at different magnifications. At a zoom of 50× no structural alterations on the underlying beech wood sample are observed, while at magnifications of 2200× or 2500× the LNPs deposited on the beech wood cell elements are clearly visible. The micrograph at 2500× from IR-LNPs-treated wood shows the sample surface in a tilted view and a crusty layer of LNPs covering a significant portion of the sample surface can be identified. At 10000× the LNPs are omnipresent on the displayed sample surfaces and a certain degree of variety depending on the original feedstock can be observed. In particular, B-LNPs and Ch-LNPs appear much smaller (average particle diameters of 210 nm and 150 nm, respectively) and with a smaller size distribution than IR-LNPs and IRNS-LNPs, the average particle diameters of which were 290 nm and 320 nm. Bigger particles might be due to different lignin structure in AL-IR and AL-IRNS with additional aromatic units resulting in higher electron densities and consecutively larger particles formed by π-stacking.

### 3.4. Color Analysis before and after Artificial Weathering Tests

**Figure 8:**
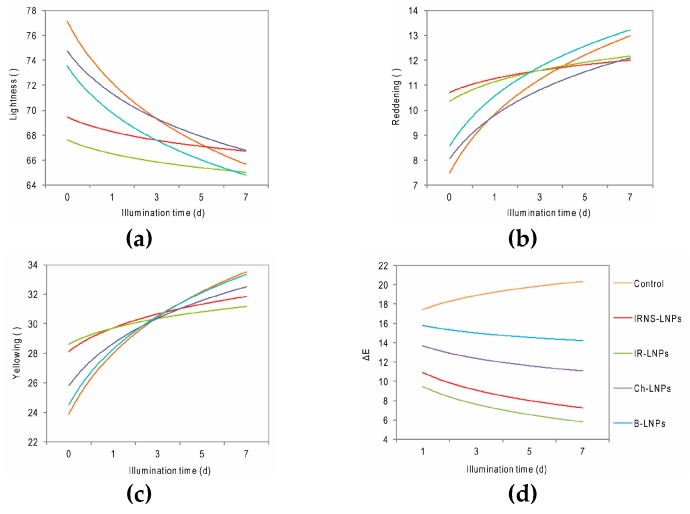
(**a**) Lightness; (**b**) Green-Red (Reddening); (**c**) Blue-Yellow (Yellowing) and (**d**) overall color difference ΔE over the treatment time of artificial weathering for the uncoated beech wood samples (control) and those coated by LNPs from beech (B-LNPs), chestnut (Ch-LNPs), Iroko (IR-LNPs) and Iroko–Norway spruce (IRNS-LNPs) in the accelerated weathering tester.

The QUV weathering tester was operated at an irradiance of 340 Wm^−2^ for a total of 168 h (7 d), giving a total UV irradiance of 57.1 kWm^−2^ over the whole length of the experiment. Jairam et al. [46] reported a UV radiation intensity of 15 Wm^−2^ in Florida, USA, which sums up to 43.8 kWm^−2^ in a year, assuming 8 hours of UV exposure per day. Considering this, the total UV exposure in our experiment corresponded to 1.3 years under real conditions in Florida, USA, and can be seen as highly significant. Smyth arrived at a total UV input of 70.4 kWm^−2^ in only 96 h exposure time using xenon arc lamps with much higher UV irradiance [20], while Jairam et al. [46] applied only a maximum of 24 h exposure time (a total of 3.2 kW/m^−2^) for the testing of polystyrene butyl acrylate films with encapsulated lignin. Considering the ΔE curves in Figure 8d, which approach a slope of 0, the authors consider the experiment duration as sufficient in order evaluate the UV protection potential of coatings based on LNPs. 

Figure 8 shows the color change over the treatment time of accelerated weathering of the beech wood samples coated with different LNPs dispersions compared to the uncoated control sample. As expected, the wood samples became darker after the initial coating by all four different LNPs with IRNS causing the highest lightness decrease of 13%. After 7 days of testing however, only the samples treated with LNPs from chestnut and IRNS were able to compensate the UV-induced darkening observed for the control sample thus maintaining higher lightness levels at the end of the treatment (Figure 8a). Regarding the changes from green to red in the CIELAB color space (Reddening), a strong initial shift towards red was evident in samples coated with LNPs from IR and IRNS. However, these samples were found to be able to successfully counteract the UV-induced modifications showing the lowest reddening at the endpoint of the treatment. Coatings made of chestnut LNPs performed in this case the best due to their ability to attenuate color changes as early as the first days of treatment (Figure 8b). Similar trends to those observed in the green-red component were found in the blue-yellow one. In particular, although the specimens coated with LNPs from IR and IRNS exhibited initially a strong shift into yellow, they were able to mitigate the changes in this color component reaching lower yellowing than the uncoated control at the end of the treatment (Figure 8c). In all three dimensions of the CIELAB color space, the samples coated by beech LNPs could not significantly compensate the color changes observed for the control sample. Figure 8d shows the overall color difference as ΔE, referred to the initial color before UV exposure, against the exposure time. Here the effect of the LNPs coatings can be observed very clear: while the uncoated control sample shows a high ΔE from the beginning which is increasing during the entire weathering simulation, the LNPs-coated samples exhibit an inverse behavior with treatment time, starting from lower ΔE, in respect to the control sample, which is decreasing towards a minimum. The lowest values for the overall color difference ΔE were determined for the samples coated with IRNS-LNPs and IR-LNPs. 

The best overall performance was observed for IRNS- and IR-LNPs-coated wood samples, qualifying IR, and IRNS mixed sawdust as valuable feedstocks for the development of new water-based wood-protection formulations. The superior performance of their respective LNPs fractions might be attributed to the presence of CP and AF units, as found by Py-GCMS and 2D NMR experiments of AL-IR and AL-IRNS, offering additional chemical sites for UV radicals scavenging.

### 3.5. SEM Analysis after Artificial Weathering Tests

**Figure 9:**
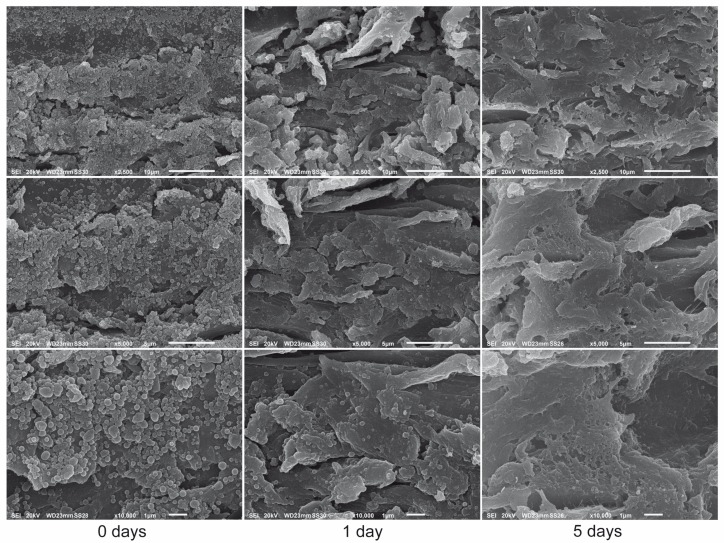
SEM micrographs of beech wood samples dip-coated with lignin nanoparticles from Iroko–Norway spruce mixed sawdust before, after 1 day and after 5 days of UV exposure in an accelerated weathering tester at different magnifications.

Morpho-structural analysis of the dip-coated samples by SEM showed that significant modifications took place in the materials over the time of the weathering treatment. As early as the first day of exposure, a residual presence of a very limited number of the nanospheres, which had retained their original shape, was evident over a background consisting of flaky and crusty aggregates with a predominantly smooth surface (Figure 9). The evident disappearance of the majority of LNPs was likely to be caused by an UV-induced coalescence process leading ultimately to their fusion. After 5 days UV exposure, very few still-intact LNPs were identified. Instead, a proper coating of the wood structure was formed where fused nanoparticles could be clearly identified at several spots. Thus, UV irradiation seemed to have formed a network of fused LNPs which, in turn, had built up a crusty protection layer of almost pure lignin on the dip-coated wood surfaces.

## 4. Conclusions

Lignin from Iroko and Iroko–Norway spruce mixed sawdust was structurally characterized by a combination of Py-GCMS and 2D HSQC NMR spectroscopy and wet-chemistry methods. The presence of residual aromatic extractives compounds in the AL fractions was strongly supported by the 2D NMR structural data as well as Py-GCMS and FTIR spectral data. The isolated lignins were successfully applied in the form of nanoparticles as a potential future wood surface protection agent. Among the four tested sawdust feedstocks (Chestnut, Beech, Iroko and Iroko–Norway spruce), Iroko and a mix of Iroko and Norway spruce were identified as promising starting materials for lignin-based wood-protection agents after automated artificial weathering experiments in a UV chamber. Aromatic extractives compounds, such as chlorophorin and alfafuran, present in the lignin macromolecule of Iroko are suspected to provide higher protection to UV irradiation and oxidation than the other tested lignins isolated from beech or chestnut. Long-term efficacy and durability of LNPs-coating need to be investigated in future experiments. 

## Figures and Tables

**Table 1:** Extractives and Klason lignin contents of the different wood samples, the respective isolated Acidolysis lignin (AL-IR, AL-IRNS) fractions and the acidolysis residues (ResIR, ResIRNS). M_w_: weight-average molar mass; M_n_: number-average molar mass. P_d_: polydispersity.

Sample	Acetone Extractives (%)	Klason Lignin (%)	Cellulose (%)	Hemi-Celluloses (%)	M_w_ (kDa)	M_n_ (kDa)	P_d_	Isolation Yield (%)
IR sawdust	5.5	29.4	40.4	11.6	-	-	-	-
ResIR	-	25.9	61.5	1.9	-	-	-	-
AL-IR	-	87.1	-	-	13.9	2.3	5.7	19.8
IRNS sawdust	3.9	29.3	40.5	14.5	-	-	-	-
ResIRNS	-	25.9	61.6	2.0	-	-	-	-
AL-IRNS	-	87.7	-	-	17.4	3.1	5.7	15.2

**Table 2:** Retention times, relative abundances, assignment of pyrolysis products to guaiacyl-type (G), syringyl-type (S) and *p*-coumaryl/hydroxyphenyl (H) units and respective S/G ratios in original Iroko sawdust (OrigIR), original mixed sawdust of Iroko–Norway spruce (OrigIRNS), respective isolated Acidolysis lignin fractions (AL-IR, AL-IRNS) and acidolysis residues (ResIR, ResIRNS). PS indicates polysaccharides origin, EX indicates extractives origin. ^*^ compounds no. 65 and 66 in Faix et al. [30].

				Iroko			Iroko–Norway Spruce		
Peak #	Pyrolysis Product	Origin	RT (min)	OrigIR (%)	AL-IR (%)	ResIR (%)	OrigIRNS (%)	AL-IRNS (%)	ResIRNS (%)
1	Phenol	H	19.35	0.8	0.4	0.4	0.7	0.6	0.5
2	Guaiacol	G	19.51	7.4	5.0	5.7	10.2	16.4	10.0
3	4-Methylphenol	H	22.35	0.0	0.3	0.0	0.0	1.3	0.0
4	4-Methylguaiacol	G	23.29	2.3	2.6	6.0	20.4	20.0	7.8
5	4-Ethylphenol	H	25.41	0.0	1.7	0.0	0.0	0.5	0.0
6	4-Ethylguaiacol	G	26.22	1.4	3.6	1.0	1.5	6.1	0.7
7	4-Vinylguaiacol	G	28.18	14.8	14.1	12.2	15.2	17.6	14.5
8	4-Vinylphenol	H	28.46	4.4	11.2	4.9	3.5	3.5	2.1
9	Eugenol	G	28.89	0.9	0.9	1.0	1.8	2.8	2.1
10	4-Propylguaiacol	G	28.95	0.0	0.0	0.0	0.7	3.7	0.0
11	Syringol	S	30.03	8.5	5.6	8.3	4.5	4.3	6.5
12	Isoeugenol (cis)	G	30.77	0.6	1.1	0.5	0.8	2.1	0.9
13	Isoeugenol (trans)	G	32.46	5.8	9.7	6.6	9.5	13.4	9.9
14	4-Methylsyringol	S	32.99	6.3	4.4	11.4	3.3	4.7	6.8
15	Vanillin	G	33.18	3.7	2.5	3.1	5.6	1.0	4.6
16	Resorcinol	Ex	34.82	1.0	11.3	0.0	0.0	1.7	0.0
17	Homovanillin	G	35.42	1.5	3.7	2.2	1.4	0.0	2.3
18	4-Ethylsyringol	S	35.55	3.9	7.4	2.0	1.4	1.8	1.5
19	Acetoguaiacone	G	35.71	2.9	3.0	2.8	2.9	0.6	3.4
20	4-Vinylsyringol	S	37.00	16.6	9.7	12.6	8.6	2.5	8.0
21	Guaiacylacetone	G	37.31	2.5	3.3	2.0	2.2	0.1	2.3
22	4-Allylsyringol	S	37.54	2.8	3.2	2.5	1.2	1.1	1.8
23	Coniferyl alcohol (cis)	G	38.59	0.6	0.0	0.0	0.6	0.0	1.2
24	4-Propenylsyringol (cis)	S	39.35	1.1	2.1	1.1	0.5	0.3	0.8
F	C_11_H_12_O_3_^*^		39.73	4.2	2.3	0.9	0.7	0.0	0.0
25	Levoglucosane	PS	40.19	1.2	0.0	5.2	3.0	0.0	11.6
26	4-Propenylsyringol (trans)	S	40.73	7.5	10.5	8.8	3.0	0.6	5.0
27	Syringaldehyde	S	41.44	1.8	1.6	2.5	0.5	0.0	1.3
28	Acetosyringone	S	43.35	2.0	1.7	1.1	0.7	0.0	1.3
29	Coniferyl alcohol (trans)	G	43.97	1.0	0.0	2.0	1.1	0.0	2.3
30	Coniferaldehyde	G	44.43	0.7	0.9	2.4	0.9	0.0	2.4
31	Syringylacetone	S	44.68	3.0	1.7	2.0	0.9	0.0	1.4
	S/G ratio			1.16	0.92	1.11	0.33	0.18	0.53

**Table 3:** Signal assignments of the ^13^C−^1^H correlation peaks in the 2D HSQC NMR spectra of the isolated lignin fractions, according to Christensen [17], del Rio et al. [33], Kim and Ralph [34], Lourenço et al. [35], Noguchi et al. [36] and Ralph et al. [39]. The labels correspond to the respective lignin substructures shown in Figure 4.

δ_C_/δ_H_ (ppm)	Assignment (label)
53.4/3.44	C*_β_*-H*_β_* in phenylcoumaran *β*-5′ substructures (B*_β_*)
53.9/3.05	C*_β_*-H*_β_* in resinol substructures *β*-*β′* (C*_β_*)
60.0/2.96‒3.82	C*_γ_*-H*_γ_* in *γ*-hydroxylated *β*-*O*-4′ substructures (A*_γ_*)
62.8/3.19	C_5_-H_5_ in *β*-d-xylopyranoside (X_5_)
63.2/3.69	C*_γ_*-H*_γ_* in phenylcoumaran *β*-5′ substructures (B*_γ_*)
64.0/4.29	C*_γ_*-H*_γ_* in *γ*-acylated *β*-*O*-4′ substructures (A*_γ′_*)
71.2/3.78 + 4.16	C*_γ_*-H*_γ_* in resinol substructures *β*-*β*′ (C*_γ_*)
72.0/4.79	C*_α_*-H*_α_* in *β*-*O*-4′ substructures (A*_α_*)
72.9/4.48	C_2_-H_2_ in 2-*O*-acetyl-*β*-d-xylopyranoside (X′_2_)
74.7/3.22	C_3_-H_3_ in *β*-d-xylopyranoside (X_3_)
75.8/4.87	C_3_-H_3_ in 3-*O*-acetyl-*β*-d-xylopyranoside (X′_3_)
77.2/3.50	_C4_-H_4_ in *β*-d-xylopyranoside (X_4_)
82.1/4.73	C*_α_*-H*_α_* in benzyl ether substructures (BE*_α_*)
82.0/4.55	C*_β_*-H*_β_* in *β*-*O*-4′ substructures (A*_β_*_(H)_) linked to a H unit
83.7/4.84	C*_α_*-H*_α_* in dibenzodioxocin substructures (D*_α_*)
83.9/5.19	C*_β_*-H*_β_* in α-oxidized (C*_α_*=O) *β*-*O*-4′ substructures (A_ox*β*_)
84.3/4.33	C*_β_*-H*_β_* in *β*-*O*-4′ substructures (A*_β_*_(G)_) linked to a G unit
85.2/4.62	C*_α_*-H*_α_* in resinol *β*-*β′* substructures (C*_α_*)
85.9/3.86	C*_β_*-H*_β_* in dibenzodioxocin substructures (D*_β_*)
86.8/4.06	C*_β_*-H*_β_* in *β*-*O*-4′ substructures (A*_β_*_(S)_) linked to a S unit
87.7/5.50	C*_α_*-H*_α_* in phenylcoumaran *β*-5′ substructures (B*_α_*)
97.9/6.91	C_7_-H_7_ in alfafuran (AF_7_)
101.6/6.22	C_4′_-H_4′_ in alfafuran (AF_4′_)
102.8/6.32	C_5_-H_5_ in chlorophorin (CP_5_)
104.1/6.64	C_2,6_-H_2,6_ in syringyl units (S_2,6_)
104.2/6.86	C_3_-H_3_ in alfafuran (AF_3_)
106.9/6.16	C_2′,6′_-H_2′,6′_ in chlorophorin (CP_2′,6′_)
107.0/7.05	C_2,6_-H_2,6_ in sinapaldehyde units (SA_2,6_)
107.0/7.25	C_2,6_-H_2,6_ in α-oxidized (C*_α_*=O) syringyl units (S′_2,6_)
107.1/6.46	C_2′,6′_-H_2′,6′_ in alfafuran (AF_2′,6′_)
107.4/6.81	C_4_-H_4_ in alfafuran (AF_4_)
107.5/6.46	C_3_-H_3_ in chlorophorin (CP_3_)
111.3/7.30	C_2_-H_2_ in ferulic acid (FA_2_)
111.3/7.01	C_2_-H_2_ in guaiacyl units (G_2_)
112.5/6.67	C_3,5_-H_3,5_ in *p*-hydroxyphenyl units (H_3,5_)
112.6/7.49	C_2_-H_2_ in oxidized guaiacyl units (G′_2_)
113.2/7.30	C_2_-H_2_ in coniferaldehyde (CA_2_)
115.9/6.96 + 6.63	C_5_-H_5_ and C_6_-H_6_ in guaiacyl units (G_5_, G_6_)
119.1/6.84	C_6_-H_6_ in guaiacyl units (G_6_)
119.5/6.84	C_6_-H_6_ in coniferaldehyde (CA_6_)
123.0/7.12	C_6_-H_6_ in ferulic acid (FA_6_)
125.0/6.74	C_7_-H_7_ in chlorophorin (CP_7_)
123.7/7.58	C_6_-H_6_ in oxidized guaiacyl units (G′_6_)
125.0/6.73	C*_β_*-H*_β_* in cinnamyl aldehyde end groups (CA*_β_*)
128.9/7.14	C_2,6_-H_2,6_ in *p*-hydroxyphenyl units (H_2,6_)
129.6/7.91	C_2,6_-H_2,6_ in *p*-coumaric acid (*p*CA_2,6_)
145.2/7.46	C*_α_*-H*_α_* in *p*-coumaric and ferulic acid (*p*CA*_α_*, FA*_α_*)
154.0/7.60	C*_α_*-H*_α_* in cinnamyl aldehyde end groups (CA*_α_*, SA*_α_*)

**Table 4:** Monomeric ratios and ratios of inter-unit linkages of the isolated acidolysis lignins from Iroko (AL-IR) and Iroko–Norway spruce (AL-IRNS), estimated on base of the cross-peaks integrals of the respective 2D NMR spectra.

Lignin Fraction	Monomeric Ratio (%)			S/G Ratio	Inter-Unit Linkages (%)					CA End Groups (%)
	S	G	H		*β*-*O*-4′	*β*-5′	*β*-*β*′	BE	DBDO	
AL-IR	44	51	5	0.86	74	15	5	4	2	2
AL-IRNS	22	75	3	0.30	71	19	4	2	4	2
